# *Tinospora crispa* Ameliorates Insulin Resistance Induced by High Fat Diet in Wistar Rats

**DOI:** 10.1155/2015/985042

**Published:** 2015-03-02

**Authors:** Mohd Nazri Abu, Suhana Samat, Norathirah Kamarapani, Fuzina Nor Hussein, Wan Iryani Wan Ismail, Hamzah Fansuri Hassan

**Affiliations:** ^1^Faculty of Health Sciences, Universiti Teknologi MARA, Puncak Alam Campus, 42300 Bandar Puncak Alam, Selangor, Malaysia; ^2^Faculty of Pharmacy, Universiti Teknologi MARA, Puncak Alam Campus, 42300 Bandar Puncak Alam, Selangor, Malaysia; ^3^Clinical BioPharmaceutics Research Group (CBRG), Brain and Neuroscience Core, Universiti Teknologi MARA, 40450 Shah Alam, Selangor Darul Ehsan, Malaysia; ^4^Faculty of Veterinary Medicine, Universiti Putra Malaysia, 43400 Serdang, Selangor, Malaysia

## Abstract

The antidiabetic properties of *Tinospora crispa*, a local herb that has been used in traditional Malay medicine and rich in antioxidant, were explored based on obesity-linked insulin resistance condition. Male Wistar rats were randomly divided into four groups, namely, the normal control (NC) which received standard rodent diet, the high fat diet (HFD) which received high fat diet only, the high fat diet treated with *T. crispa* (HFDTC), and the high fat diet treated with orlistat (HFDO). After sixteen weeks of treatment, blood and organs were harvested for analyses. Results showed that *T. crispa* significantly (*p* < 0.05) reduced the body weight (41.14 ± 1.40%), adiposity index serum levels (4.910 ± 0.80%), aspartate aminotransferase (AST: 161 ± 4.71 U/L), alanine aminotransferase (ALT: 100.95 ± 3.10 U/L), total cholesterol (TC: 18.55 ± 0.26 mmol/L), triglycerides (TG: 3.70 ± 0.11 mmol/L), blood glucose (8.50 ± 0.30 mmo/L), resistin (0.74 ± 0.20 ng/mL), and leptin (17.428 ± 1.50 ng/mL) hormones in HFDTC group. The insulin (1.65 ± 0.07 pg/mL) and C-peptide (136.48 pmol/L) hormones were slightly decreased but within normal range. The histological results showed unharmed and intact liver tissues in HFDTC group. As a conclusion, *T. crispa* ameliorates insulin resistance-associated with obesity in Wistar rats fed with high fat diet.

## 1. Introduction

Obesity is a leading contributor to global metabolic diseases. Around 3.4 million adults became overweight or obese each year and more than 40 million children under age of five were overweight in 2012 worldwide [1]. Consumption of high amount of fat and calories in a diet leads to obesity. The ability of obesity to engender insulin resistance is linked to a wide array of pathophysiologic sequels particularly hyperinsulinemia and type 2 diabetes mellitus. The insulin resistance condition interferes with glucose utilization in the liver and skeletal muscles thus reducing glycogen storage and affects glucose homeostasis. Interference in glucose homeostasis interrupts extracellular and intracellular glucose concentrations, consequently amplifying insulin production by the pancreas, leading to hyperinsulinemia. Prolong hyperinsulinemia condition promotes oxidative stress due to increased production of reactive oxygen species (ROS). Increment in ROS and oxidative stress is a key in triggering the progression of metabolic complications such as diabetic nephropathy [2]. The uncontrolled phenomenon can lead to metabolic diseases such as hypertension, heart disease, and cancer [3]. Insulin resistance also enhances the accumulation and infiltration of adipose tissue by inflammatory cells, subsequently causing inflammation, and exacerbates the complications. Losing weight through drug therapy requires follow-up diagnosis due to possible adverse effects of synthetic chemical drugs. Therefore, it is important to find alternative treatments for metabolic diseases related to obesity and insulin resistance, especially from nature-based products.* Tinospora crispa*, a herbal plant which is found rich in antioxidants and possesses antidiabetic properties, has attracted researchers to investigate its possible ameliorative effects on induced insulin resistance in obese subjects [4, 5]. Therefore, this study aims to observe the effects of* T. crispa* in ameliorating insulin resistance induced in obese Wistar rats.

## 2. Methods

### 2.1. Sample Collection

*T. crispa* sample was purchased in powdered form from the Brotocafe Manufacturing Sdn Bhd, Malaysia (962191-W). The powders were dissolved in 1 : 1 g/mL warm distilled water (60–70°C) prior to use [6].

### 2.2. Animal Care

Male Wistar rats, aged seven weeks old, were obtained from Laboratory Animal Facility and Management, (LAFAM), Universiti Teknologi MARA (UiTM), Puncak Alam, Malaysia. All animal-related experiments were carried out according to the protocol approved by the Research Committee on the Ethical Use of Animals (UiTM Care), reference number: 18/2013. Animals were housed as one rat per cage at the ambient temperature of 25 ± 2°C and 40–65% relative humidity, with 12-hour light/dark cycle [7]. The rats were given standard rodent diet (11.8 kcal% fat), from Rodent Diet Speciality Feeds, Glen Forrest, Australia and distilled water was provided* ad libitum* for 1 week.

### 2.3. Induction of Insulin Resistance

After acclimatization for one week, animals were randomly divided into four different groups namely, the normal control (NC) which received standard rodent diet, the high fat diet (HFD) which received high fat diet only, the high fat diet treated with* T. crispa *(HFDTC), and the high fat diet treated with orlistat (HFDO). For the first eight weeks, three groups (HFD, HFDTC and HFDO) were fed with purified high fat diet daily to increase rapid weight gain and obesity thus inducing insulin resistance. For the subsequent eight weeks, HFDTC and HFDO were given oral administration of* T. crispa* and orlistat at 100 mg/kg body weight/day, respectively. Meanwhile, NC and HFD groups were continued with their previous diets. At the end of the treatment period, all of the rats were fasted for 16 hours and sacrificed by decapitation. Blood samples were collected through cardiac puncture and left to clot without anticoagulants in plain serum separating tube (SST).

### 2.4. Body Weight and Meal Pattern Analysis

The body weight (BW) of each rat was recorded once per week and the differences in BW were noted. In the meal pattern analysis, the amount of food and water consumed was measured weekly by subtracting from the quantity of food and water supplied initially. The food efficiency was calculated once, at the end of the study. The total number of kilocalories that was consumed by each rat was determined by multiplying the caloric content of 1 gram of each diet by the total quantity of food eaten.

### 2.5. Biochemical Analysis

Blood samples were freshly collected through cardiac puncture, and the samples were stored in plain SST tubes for serum biochemical assay. The blood samples were centrifuged at 3000 rpm at 4°C for 15 minutes. The clear serum obtained was separated and labeled for liver function tests (LFT), namely, aspartate aminotransferase (AST) and alanine aminotransaminase (ALT), renal function test (RFT), namely, urea and creatinine, fasting blood sugar (FBS) and serum lipid profile (LP), namely, triglycerides (TG) and total cholesterol. Biochemical tests were performed using ILAB 300 Plus Clinical Chemistry Analyzer, Milano, Italy. Serum leptin, resistin, insulin hormone (USCN Life Science and Technology, Wuhan, China), C-peptide (Mercodia AB, Uppsala, Sweden), and serum levels were determined through ELISA method by referring to the respective manufacturer's instructions.

### 2.6. Anthropometrical and Adiposity Index Determinations

The body weight and body length were determined by anthropometrical parameters:

(1) $$\begin{array}{c} \text{Body}\;\;\text{mass}\;\;\text{index}\;\;\left(\text{BMI}\right)=\frac{\text{body}\;\;\text{weight}\;\left(\text{g}\right)}{{\text{length}}^{2}\;\left({\text{cm}}^{2}\right)},\\ \text{Lee's}\;\;\text{index}=\frac{\text{cube}\;\;\text{root}\;\;\text{of}\;\;\text{body}\;\;\text{weight}\left(\text{g}\right)}{\text{nose}\;\;\text{to}\;\;\text{anus}\;\;\text{length}\left(\text{cm}\right)}.\; \end{array}$$

Adiposity index was determined by the sum of epididymal, visceral, and retroperitoneal fat weights divided by body weight × 100 and expressed as adiposity percentage (% AI).

### 2.7. Histological Evaluation

A comprehensive gross observation was carried out and histological examination of liver toxicity was performed. Any signs of abnormality or presence of lesions on the organ was observed following administration of* T. crispa* and high fat diet intake [8, 9]. The organs were then carefully dissected, cleaned of any fats, and weighed. The relative organ weight (ROW) of each organ was then calculated according to the following equation:

(2) $$\begin{array}{c} \text{ROW}=\frac{\left(\text{absolute}\;\;\text{organ}\;\;\text{weight}\;\;\left(\text{g}\right)\times 100\right)}{\text{body}\;\;\text{weight}\;\;\text{of}\;\;\text{rat}\;\;\mathrm{on}\;\;\text{sacrifice}\;\;\text{day}\;\;\left(\text{g}\right)}. \end{array}$$

Each organ was then preserved in 10% buffered formalin for subsequent histopathological examination. The tissues were embedded in paraffin, sectioned in 4-5 *μ*m thick using the rotary microtome, stained with hematoxylin and eosin, and examined microscopically [8, 9].

### 2.8. Statistical Analysis

Results were expressed as mean ± standard error mean (SEM). Statistical significance was determined by one-way analysis of variance (ANOVA). Values with a confidence level of *p* < 0.05 were considered significant.

## 3. Results

### 3.1. Body Weight and Meal Pattern

Total food intake and energy efficiency for HFDTC group were higher compared to the HFD group (Table 1). However, percentage of body weight in HFDTC group showed a significantly decreased (41.14%) compared to HFD (47.57%) group. No significant differences in calorie consumptions and value of energy efficiency were observed in HFDO compared to NC group.

### 3.2. Serum Level of Liver and Renal Enzymes

Hepatic serum (AST and ALT) and renal serum levels (total protein, creatinine, and urea) were significantly reduced in the HFDTC compared to HFD group (Table 2). However, total protein and creatinine levels (130.75 mmol/L and 105.01 mmol/L) in HFDO group showed significant increases compared to NC (84.25 mmol/L and 76.51 mmol/L) group, respectively (Table 3).

### 3.3. Serum Level of Glucose, Cholesterol, and Triglycerides

Levels of glucose, cholesterol, and triglycerides were shown in Table 4. Serum glucose, cholesterol, and triglycerides levels were significantly reduced in HFDTC compared to HFD groups. HFDTC exhibited a comparable result to NC group.

### 3.4. Level of Adipocytokines (Resistin and Leptin), Insulin, and C-Peptide

Resistin, leptin, insulin, and C-peptide concentrations were markedly increased in HFD group compared to the others (Table 5). However, it was the direct opposite in HFDTC, where significant decreases were observed in all tests compared to HFD group. Resistin and insulin levels in HFDO group remained low.

### 3.5. Anthropometrical and Adiposity Index

The rats fed with high fat diet (HFD) gave significantly higher BMI and Lee's index compared to NC group (Table 6). However, treatment with TC (HFDTC) showed significant reductions (25.00 g) in adipose tissue weight compared to HFD (32.28 g) groups. Moreover, HFDTC group showed significantly decreased BMI, Lee's index, and adiposity index compared to HFD group.

### 3.6. Relative Organ Weight and Histopathology Evaluation

**Figure 1:**
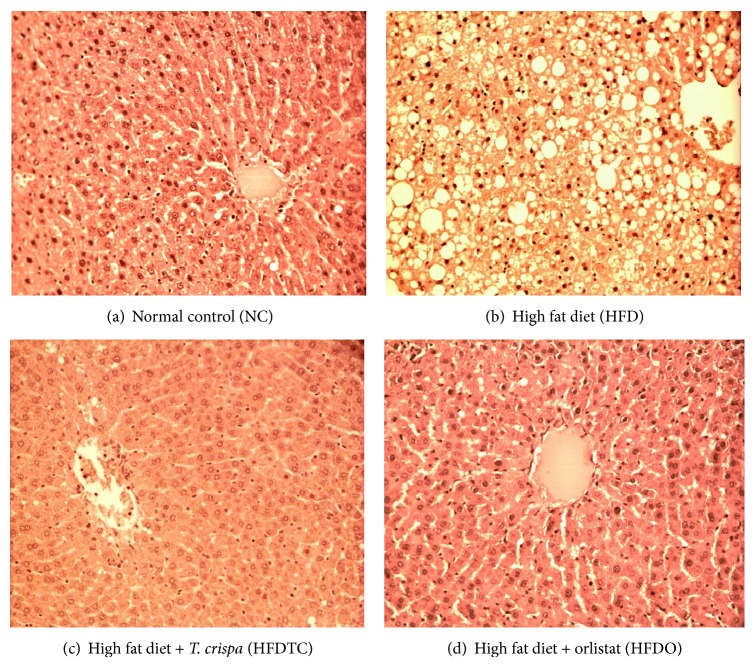
Pictomicrographs of the liver of NC (a) rats and insulin resistant male Wistar rats induced with high fat diet before (b) and after being treated with* T. crispa* (c) and orlistat (d) (H&E staining ×20). The HFD group (b) showed severe degrees of micro- and macrovesicular steatosis and severe hepatocellular ballooning while the rest ((a), (b), and (c)) showed normal hepatocytes with intact morphologies.

The absolute and relative organ weights (ROW) of the isolated hearts, spleens, kidneys, lungs, and livers from the groups were recorded and calculated (Table 7). Gross necropsy findings did not reveal changes in any of the organs examined (Figure 1). The relative organ weights for liver, heart, and lung recorded at the end of the study showed a significant decrease for rats in HFDTC group compared to the rats in HFD group.

## 4. Discussions

Our study showed that rats fed with high fat diet demonstrated pathophysiology of the insulin resistance condition. The development of glucose intolerance with hyperglycemia, hyperinsulinemia, and markedly increased body weight was observed in long-term consumptions of high fat diet. The rats fed with high caloric food were prone to develop weight gain and causing excessive deposition of fats in the body as seen in the results from the HFD group (Table 1). For that reason, intake of high fat and high caloric foods could become major factors that contribute to obesity [9]. To render* in vivo* insulin resistant obese condition, the male Wistar rats were subjected to a high fat diet regime for eight weeks. The insulin resistant obese condition was developed in week 4, along with hyperglycemia, hyperinsulinemia, and increased C-peptide, together with increased body weight, fat weight, and adiposity index [10].

On the contrary, the results showed that energy efficiency was higher in the high fat diet rats treated with* T. crispa* (HFDTC) compared to HFD group (Table 1). There was a significant correlation between energy efficiency and the decrease in body weight detected in HFDTC group. Together, these results indicate the synergistic effect of* T. crispa* on the lipid metabolism of the rats. Previous studies reported that concentration used in this study (100 mg/kg/day continuously for 28 days) did not cause any dose-related changes that can lead to the breakdown of bodily functions [6, 8]. Interestingly, the concentration of* T. crispa* used in this experiment resulted in significant decrease in body weight compared to the HFD group.

The hepatoprotective potential of* T. crispa* is demonstrated by the significantly lower levels of AST and ALT in the HFDTC group (Table 2) compared to their levels in the HFD and HFDO groups. These results are consistent with other studies [10, 11]. Furthermore, these results are supported by the findings of intact liver tissues in HFDTC group's histological study (Figure 1(c)). Collectively, these findings strongly suggest the potential of* T. crispa* in controlling liver damage caused by high fat diets. However, the underlying mechanism of action for these effects is still unclear. The presence of antioxidants like flavonoids and phenolics in* T. crispa* may help to reduce inflammation in the body due to their cytoprotective and anti-inflammatory actions [9, 11–13]. Kidney and spleen showed no significant changes in relative organ weight (ROW) in the HFDTC group (Table 7). Furthermore, histological investigation showed no pathological changes in the livers of HFDTC rats (Figure 1(c)). Histological sections of livers from the HFDTC groups showed intact liver parenchyma with the central vein clearly seen along with bile ducts and hepatic arteries. The hepatocytes were also neatly arranged with the sinusoids radiating from the central vein (Figure 1(c)). Conversely, rats fed with HFD for sixteen weeks showed incidences of hepatocytes hypertrophy, fat deposition, and infiltration of a mixed population of inflammatory cell as well as ballooning degeneration of hepatocytes characterized by cell swelling with empty intracellular content (Figure 1(b)), indicating cell necrosis in their liver.

Normal kidney function observed in HFDTC group (Table 3) suggests that* T. crispa* is safe for consumption at the experimental dose. The decrease in HFDTC lipid profile (Table 4) demonstrated the hypolipidemic effect of* T. crispa*, probably through inhibition of the intestinal absorption of cholesterol and triglycerides, thus reducing the accumulation of fats in the body.

There were no other specific mechanisms of action of* T. crispa'*s antioxidants properties in reducing the body weight on treated rats [14] but common alkaloid; berberine compound was suggested as an antiobesity agent due to the activity of AMP-activated protein kinase (AMPK) in the peripheral tissues [13]. Berberine was proposed to elevate the fatty acid oxidation through activation of AMPK in muscle cells and thus improving lipid metabolism in the body which was frequently related to hyperlipidemia and fatty liver in obese patients [5]. Furthermore, the effect of* T. crispa* on diabetic mice revealed activation of insulin receptor-AKT-GLUT2 expression and insulin sensitivity enhancement by borapetosides A, B, and C which contributed to the hypoglycemic effect* in vivo* [15–17].

Findings in an earlier study [18] supported that leptin was a factor that could be responsible for energy homeostasis in the body involving body fat mass and total amounts of food consumed [14]. Increase secretion of leptin from adipose tissues in obese state should suppress the appetite and consequently food intake. Leptin aids in improving the energy expenditure and prevents the accumulation of fat in the body especially subcutaneous tissues and abdominal adipose tissues [19]. In the present study, however, increased level of leptin (Table 5) as found in HFD group was proposed to develop leptin resistance rather than insufficiency of leptin, which result in the increment in their appetite and food intake [16]. In contrast, the HFDTC showed markedly decreased leptin level (Table 5), similar to orlistat treated rats (HFDO). In general, the mechanism of action of orlistat is by facilitating the decrease in the absorption of fat in gastrointestinal tract, thus preventing excessive fat deposition in the body. Orlistat has been effectively used clinically for overweight treatment even though there are various adverse effects for long-term consumption [20, 21].

The results of* T. crispa* administration on rats in HFDTC group showed promises in countering the effect of insulin resistance and as an antiobesity agent. This is evidenced by the markedly reduced levels of resistin-leptin, thus amplifying insulin and C-peptide hormones (Table 5).* T. crispa* treatment has lowered the secretion of insulin hormones in HFDTC rats compared to rats in NC group, countering the hyperinsulinemia effects which is the main characteristic of insulin resistance [4, 5, 16].

The administration of* T. crispa* in HFDTC group has significantly lowered glucose level (Table 4) compared to HFD group. This elucidates the effectiveness of this herb in reducing the glucose level in obese rat with high fat intake. It is proposed that the hyperglycemia condition due to the high caloric diet intake in HFD has altered the pancreatic-*β* cells secretion, resulting in insulin resistance development [15]. Perhaps, the presence of  borapetosides A, B, and C in* T. crispa* which regulates glucose uptake in the cells and peripheral tissues utilization [16] could counter this effect. In addition, other studies have demonstrated the consistent hypoglycemic effect of* T. crispa* in diabetic-induced rats [4, 5].

## 5. Conclusion

In conclusion, through oral administration of* Tinospora crispa* crude extract to insulin resistant obese Wistar rats induced by high-fat diet conducted in this study has shown that the herb exhibited antidiabetic, antihypercholesterolemic, and hepatoprotective effects. These results suggest that long-term consumption of* T. crispa* may be useful in treating obesity in patients with insulin resistance and diabetes mellitus conditions.

## Figures and Tables

**Table 1:** Effect of *T. crispa* on body weight (BW), calories (kJ), and energy efficiency.

Parameter groups	Percent body weight (%, BW)	Total food intake (g)	Calories (kJ)	Energy efficiency
Normal control (NC)	35.63 ± 1.79	2082.30 ± 18.60	27.81 ± 4.21	0.029 ± 0.0004
High fat diet (HFD)	47.57 ± 1.28	2202.21 ± 23.30	27.17 ± 7.47	0.023 ± 0.0010
High fat diet + *T. crispa* (HFDTC)	41.14 ± 1.40^*^	2211.82 ± 16.51	27.59 ± 8.72	0.028 ± 0.0005^*^
High fat diet + orlistat (HFDO)	40.26 ± 1.06^*^	2103.00 ± 19.24	26.90 ± 3.62	0.024 ± 0.0003

Means ± SEM with “∗” in the same column are significant at *p* < 0.05 compared to HFD group using one-way ANOVA test. *n* = 5 rats/group.

**Table 2:** Effect of *T. crispa* on levels of aspartate aminotransferase (AST) and alanine aminotransferase (ALT) liver enzymes in rats.

Parameter groups	AST (U/L)	ALT (U/L)
Normal control (NC)	154.51 ± 2.10	92.73 ± 1.06
High fat diet (HFD)	218.10 ± 2.94	136.00 ± 2.44
High fat diet + *T. crispa * (HFDTC)	161.25 ± 4.71^*^	100.95 ± 3.10^*^
High fat diet + orlistat (HFDO)	187.50 ± 3.00^*^	115.00 ± 4.35^*^

Means ± SEM with “∗” in the same column are significant at *p* < 0.05 compared to HFD group using one-way ANOVA test. *n* = 5 rats/group.

**Table 3:** Effect of *T. crispa* on total protein, creatinine, and urea.

Parameter groups	Total protein (mmol/L)	Creatinine (mmol/L)	Urea (mmol/L)
Control (NC)	84.25 ± 1.58	76.51 ± 1.26	12.11 ± 1.10
High fat diet (HFD)	196.75 ± 2.36	92.00 ± 2.71	18.35 ± 0.50
High fat diet + *T. crispa *(HFDTC)	99.43 ± 1.33^*^	77.75 ± 3.28^*^	14.75 ± 1.03^*^
High fat diet + orlistat (HFDO)	130.75 ± 1.48^*^	105.01 ± 3.00^*^	15.25 ± 1.03

Means ± SEM with “∗” in the same column are significant at *p* < 0.05 compared to HFD group using one-way ANOVA test. *n* = 5 rats/group.

**Table 4:** Effect of *T. crispa* (TC) on glucose, cholesterol, and triglycerides (TG).

Parameter groups	Glucose (mmol/L)	Cholesterol (mmol/L)	Triglycerides (mmol/L)
Control (NC)	7.25 ± 0.48	14.03 ± 0.50	2.58 ± 0.10
High fat diet (HFD)	13.75 ± 0.25	23.38 ± 0.23	4.65 ± 0.10
High fat diet + *T. crispa *(HFDTC)	8.50 ± 0.30^*^	18.55 ± 0.26^*^	3.70 ± 0.11^*^
High fat diet + orlistat (HFDO)	11.50 ± 0.30^*^	13.00 ± 0.41^*^	3.58 ± 0.03^*^

Means ± SEM with “∗” in the same column are significant at *p* < 0.05 compared to HFD group using one-way ANOVA test. *n* = 5 rats/group.

**Table 5:** Effect of *T. crispa* on resistin, leptin, insulin, and C-peptide.

Parameter groups	Resistin (ng/mL)	Leptin (ng/mL)	Insulin (pg/mL)	C-peptide (pmol/L)
Control (NC)	0.99 ± 0.10	18.721 ± 2.14	1.59 ± 0.07	161.50 ± 5.42
High fat diet (HFD)	1.13 ± 0.20	14.725 ± 1.11	2.27 ± 0.08	301.66 ± 6.8
High fat diet + *T. crispa *(HFDTC)	0.74 ± 0.20^*^	17.428 ± 1.50	1.65 ± 0.07^*^	136.48 ± 4.1^*^
High fat diet + orlistat (HFDO)	0.84 ± 0.20	22.620 ± 1.74	1.98 ± 0.07	173.92 ± 5.11^*^

Means ± SEM with “∗” in the same column are significant at *p* < 0.05 compared to HFD group using one-way ANOVA test. *n* = 5 rats/group.

**Table 6:** Effect of *T. crispa* on body mass index (BMI), Lee's Index, adiposity Index (AI), and total fat pads.

Parameter groups	Body mass index (BMI)	Lee's index	Total fat pads	Adiposity index, AI (%)
Normal control (NC)	0.641 ± 0.01	0.293 ± 0.002	18.378 ± 2.92	3.312 ± 0.40
High fat diet (HFD)	0.836 ± 0.02	0.322 ± 0.005	32.278 ± 4.17	6.204 ± 0.30
High fat diet + *T. crispa *(HFDTC)	0.760 ± 0.02^*^	0.309 ± 0.001^*^	25.002 ± 4.40^*^	4.910 ± 0.80^*^
High fat diet + orlistat (HFDO)	0.783 ± 0.02^*^	0.308 ± 0.003^*^	29.216 ± 2.87^*^	5.120 ± 0.41^*^

Means ± SEM with “∗” in the same column are significant at *p* < 0.05 compared to HFD group using one-way ANOVA test. *n* = 5 rats/group.

**Table 7:** Effect of *T. crispa* on relative organ weight (ROW) of liver, kidney, lung, spleen, and heart.

Parameter groups	Liver	Kidney	Lung	Spleen	Heart
Normal control (NC)	2.404 ± 0.05	0.546 ± 0.03	0.305 ± 0.01	0.173 ± 0.02	0.242 ± 0.03
High fat diet (HFD)	3.101 ± 0.14	0.520 ± 0.06	0.377 ± 0.02	0.161 ± 0.02	0.319 ± 0.02
High fat diet + *T. crispa *(HFDTC)	2.510 ± 0.08^*^	0.520 ± 0.03	0.317 ± 0.04^*^	0.167 ± 0.01	0.292 ± 0.02^*^
High fat diet + orlistat (HFDO)	2.325 ± 0.02^*^	0.4361 ± 0.03^*^	0.296 ± 2.87^*^	0.172 ± 0.02^*^	0.303 ± 0.01^*^

Means ± SEM with “∗” in the same column are significant at *p* < 0.05 compared to HFD group using one-way ANOVA test. *n* = 5 rats/group.
